# Remote Training of Neurointerventions by Audiovisual Streaming: Experiences from the European ESMINT-EYMINT E-Fellowship Program

**DOI:** 10.1007/s00062-022-01192-9

**Published:** 2022-07-13

**Authors:** Uta Hanning, Matthias Bechstein, Johannes Kaesmacher, Grégoire Boulouis, René Chapot, Tommy Andersson, Edoardo Boccardi, Marios Psychogios, Christophe Cognard, Marta de Dios Lascuevas, Marta Rodrigues, Isabel Rodriguez Caamaño, Sergios Gargalas, Davide Simonato, Vedran Zupancic, Cornelia Daller, Lukas Meyer, Gabriel Broocks, Helena Guerreiro, Jens Fiehler, Mario Martínez-Galdamez, Vladimir Kalousek

**Affiliations:** 1 Department of Diagnostic and Interventional Neuroradiology, University Medical Center Hamburg-Eppendorf, Martinistraße 52, 20246 Hamburg, Germany; 2 Department of Neurology, Institute of Diagnostic and Interventional Neuroradiology, Inselspital, Bern University Hospital, Bern, Switzerland; 3 Diagnostic and Interventional Neuroradiology Department, Institut national de la santé et de la recherche médicale (INSERM) Team 1253 iBrain, Tours University Hospital, Tours, Centre Val de Loire Region France; 4 Department of Neuroradiology, Alfried Krupp Krankenhaus, Essen, Germany; 5 Department of Neuroradiology, Karolinska University Hospital and Clinical Neuroscience, Karolinska Institute, Stockholm, Sweden; 6 Department of Medical Imaging, AZ Groeninge, Kortrijk, Belgium; 7 Department of Diagnostic and Interventional Neuroradiology, Niguarda Hospital, Milan, Italy; 8 Division of Neuroradiology, Clinic of Radiology and Nuclear Medicine, Basel University Hospital, Basel, Switzerland; 9 Neuroradiology Department, Pierre-Paul-Riquet/Purpan University Hospital, Toulouse, France; 10 Neuroradiology, Vall d’Hebron Hospital Universitari, Barcelona, Spain; 11 Neuroradiology Department, Centro Hospitalar de Vila Nova de Gaia/Espinho, Vila Nova de Gaia, Portugal; 12 Radiology Department, Hospital Universitari de Bellvitge, Barcelona, Spain; 13 Diagnostic and Interventional Neuroradiology Department, John Radcliffe Hospital, Oxford, UK; 14 Interventional Neuroradiology Department, University Hospital of Padua, Padua, Italy; 15 Department of Radiology, Clinical Hospital Center Sestre Milosrdnice, Zagreb, Croatia; 16 Department of Neurosurgery, Paracelsus Medical University Salzburg, Salzburg, Austria; 17 Interventional Neuroradiology/Endovascular Neurosurgery, Hospital Clínico Universitario de Valladolid, Valladolid, Spain

**Keywords:** Telemedicine, Teleproctoring, Teleobservership, Neuroendovascular training, Stroke

## Abstract

**Background:**

Remote access of trainees to training centers via video streaming (tele-observership, e‑fellowship) emerges as an alternative to acquire knowledge in endovascular interventions. Situational awareness is a summary term that is also used in surgical procedures for perceiving and understanding the situation and projecting what will happen next. A high situational awareness would serve as prerequisite for meaningful learning success during tele-observerships. We hypothesized that live perception of the angiographical procedures using streaming technology is feasible and sufficient to gain useful situational awareness of the procedure.

**Methods:**

During a European tele-observership organized by the European Society of Minimally Invasive Neurological Therapy (ESMINT) and its trainee association (EYMINT), a total of six neurointerventional fellows in five countries observed live cases performed by experienced neurointerventionalists (mentors) in six different high-volume neurovascular centers across Europe equipped with live-streaming technology (Tegus Medical, Hamburg, Germany). Cases were prospectively evaluated during a 12-month period, followed by a final questionnaire after completion of the course.

**Results:**

A total of 102/161 (63%) cases with a 1:1 allocation of fellow and mentor were evaluated during a 12-month period. Most frequent conditions were ischemic stroke (27.5%), followed by embolization of unruptured aneurysms (25.5%) and arteriovenous malformations (AVMs) (15.7%). A high level of situational awareness was reported by fellows in 75.5% of all cases. After finishing the program, the general improvement of neurointerventional knowledge was evaluated to be extensive (1/6 fellows), substantial (3/6), and moderate (2/6). The specific fields of improvement were procedural knowledge (6/6 fellows), technical knowledge (3/6) and complication management (2/6).

**Conclusion:**

Online streaming technology facilitates location-independent training of complex neurointerventional procedures through high levels of situational awareness and can therefore supplement live hands-on-training. In addition, it leads to a training effect for fellows with a perceived improvement of their neurointerventional knowledge.

**Supplementary Information:**

The online version of this article (10.1007/s00062-022-01192-9) contains supplementary material, which is available to authorized users.

## Introduction

Continuous live observation of various interventional procedures is a key element of neurointerventional training and a prerequisite for safely performing stand-alone interventions on patients in a clinical environment. A major challenge for young interventionalists is to reach an adequate volume of observed cases, particularly in the face of steadily evolving techniques, and abundance of devices on the market [1, 2]. The coronavirus disease 2019 (COVID-19) pandemic has further complicated training observerships, either due to travel restrictions making fellowships in high-volume centers unfeasible, or due to limited availability of on-site training and practical courses by dedicated specialists. Telemedicine with live remote broadcast of surgical procedures is a potential key driver of change in this context and has started to transform the training environment in different surgical subspecialities, but especially in the neurointerventional environment [3–7]. Several technical solutions enable live audiovisual on-demand streaming from the angiography suite to selected viewers, who can choose from different points of viewing onto the patient table and angiography monitor, and simultaneously talk with the treating interventionalist [1, 8–10]. While this technology has so far been primarily used to connect a remote specialist with the purpose of expert supervision or virtual guidance, the technology also vice versa allows remote fellows to observe cases performed by a specialist at a dedicated center.

Situational awareness has been historically used to describe the level of focus of aircraft pilots and is defined as “the perception of elements of the environment within a volume of time and space, the comprehension of their meaning and the projection of their status in the near future” [11]. It is considered a critical non-technical component of decision-making during complex medical and non-medical procedures. Aim of this study was to evaluate the feasibility of a telestream set-up (Tegus Medical, Hamburg, Germany) as means to expand the volume of observed cases for trainee interventionalists. We hypothesized that audiovisual streaming technology is sufficient to gain meaningful situational awareness of neurovascular procedures, as evaluated by the remote observer.

## Methods

The European Society of Minimally Invasive Neurological Therapy (ESMINT) and its trainee association (EYMINT) initiated the e‑fellowship program, which recruited expert neurointerventionalists (mentors) at six different high-volume centers across Europe to stream and comment on interventions they performed to selected trainee neurointerventionalists (e-fellows, tele-observers). The cases were presented in a fixed 1:1 allocation of a mentor and a tele-observer for a period of 6–12 months in order to enable a familiar environment for both parties and minimize possible distraction of the treating interventionalist by an alternating audience. Selected cases of particular interest were made available to all trainee interventionalists at thediscretion of the mentor. Cases were prospectively evaluated by the fellow, followed by a final questionnaire after completion of the course for both the fellow and mentor.

### Telestream Technology, Set-up and Data Privacy

For the purpose of the e‑fellowship, a dedicated live telestream set-up commercially available from Tegus Medical GmbH (Hamburg, Germany) was installed in six high-volume neurovascular centers in six different European countries. The centers were selected by the EYMINT committee members primarily based on neurointerventional practice and the local availability of a neurointerventional expert with experience as course instructor. The telestream set-up had been specifically developed for low-latency high-resolution streaming with emphasis on stable image transmission quality and was tested successfully in simulated and real-world patient cases [1, 3]. The hardware set-up consisted of a 360° rotatable and 180° tiltable high-definition network camera, placed on a freely movable tripod. The unit required a broadband internet access through the hospital provider, but not any structural modifications inside the angiography suite or a connection to the angiography unit itself. Camera functions, i.e. positioning of the field of view, focus and zoom, were controlled remotely by the e‑fellow from a work or home computer using a web-based user interface. Access to the interface was password secured and required initial clearance from the treating interventionalist in each case. Each participating center obtained ethical approval according to their local protocol to share retrospective and fully anonymized data. The treating interventionalist and the e‑fellow communicated with each other using the voicestream function of the set-up and headsets. All data transfer was encrypted. The data privacy protocol further included that e‑fellows did not receive any information about the patient’s identity. In addition, patient names on the angiography monitors were either anonymized by default or physically blinded. Patients had given informed consent for the streaming whenever possible.

### Requirements for E-fellowship Participants and Selection Process

Selection of e‑fellows was based on an application process managed by EYMINT. Minimum requirements for applicants were 1) possession of a medical license to practice neurointervention and 2) at least 1 year experience in neurointervention with ability to independently perform diagnostic angiographies. Each mentor chose a fellow from a set of three preselected applicants. The proposed curriculum of telestreamed cases during a 6-month fellowship is depicted in Table 1.

**Table 1:** Proposed curriculum of a 6-month telestream fellowship for trainee interventionalists

Type of intervention	Required minimum number of observed cases
Acute ischemic stroke (recanalization)	10
Intracranial aneurysm (embolization, flow diversion etc.)	10
Extracranial/intracranial vessel stenosis (angioplasty, stenting)	2
Vascular malformations (AVM, dAVF embolization)	Dependent on case frequency at assigned neurovascular center

*AVM* arteriovenous malformation, *dAVF* dural arteriovenous fistula

### Case Selection and Evaluation

Mentors decided independently which cases to telestream to their respective fellows. Aim was to involve the e‑fellows as early as possible in the clinical indication setting, preferably with a case briefing by video conference prior to the intervention. After the patient was positioned in the angiography suite and patient identifying information on the angiography monitor covered, the mentor enabled the telestream set-up. The fellow then joined the session through the web-based user interface from a conventional computer. The system set-up has been described in the literature including the control options for the remote observer, i.e. the option to freely move the camera’s field of view and use of a zoom function [1, 3]. Continuous one-to-one communication between the mentor and fellow was enabled by headsets and voicestream. After completion of the intervention, a debriefing was performed in most cases to discuss procedural details and recapitulate essential learning points. The fellows were then asked to fill in a standardized anonymous online questionnaire. The collected data included questions about the procedure type, technical feasibility of the set-up, a subjective estimation of the learning progress and evaluation of the perceived situational awareness. A full list of the questions is included in Supplement A. After completion of the course fellows were again asked to fill in a general questionnaire concerning the overall estimation of the learning progress (post-fellowship evaluation). Mentors were also asked to evaluate the course with emphasis on technical feasibility of the telestream set-up.

### Joint Sessions

In addition to the single sessions with 1:1 assignment of mentor and fellow, the course program included occasional joint sessions for a larger audience. In this case, a procedure of special interest (i.e. embolization of a ruptured blister aneurysm) was streamed to all fellows at the discretion of the treating interventionalist. The post-fellowship evaluation included a comparison of the learning progress achieved through single versus joint sessions.

### Statistical Analysis

For categorical data, absolute and relative frequencies are given. Mean values are depicted with their standard deviation. All analyses were performed using IBM SPSS Statistics, Version 26.0 (IBM, Armonk, NY, USA).

## Results

During the duration of the fellowship from May 2020 to April 2021, a total of 161 cases were transmitted. Online evaluation using the standardized questionnaire was registered in 102 cases (63%). Types of interventions and procedural characteristics are depicted in Table 2.

**Table 2:** Overview of evaluated telestreamed cases and procedures

Cases (*n*)	Procedures (*n*)
Unruptured aneurysm (26)	Coil Embolization (2)
	Balloon/Stent-assisted Coil Embolization (10)
	Flow Diversion (11)
	Intrasaccular Device (3)
Ruptured aneurysm (8)	Coil Embolization (4)
	Balloon‑/Stent-assisted Coil Embolization (2)
	Flow Diversion (2)
Arteriovenous malformation (16)	Embolization (16)
Dural arteriovenous fistula (14)	Embolization (14)
Acute ischemic stroke (28)	Stent-Retriever (24)
	Aspiration alone (1)
	Thrombectomy + Carotid Stent/Angioplasty (3)
Carotid artery stenosis (5)	Carotid Stent/Angioplasty (5)
Other (5)	Vasospasm—Angioplasty (2)
	Diagnostic Intracranial Angiogram (2)
	Spinal Angiogram (1)

Cases (evaluated) *n* = 102

Most frequent interventions were thrombectomies in acute ischemic stroke (27.5%), followed by embolization of unruptured aneurysms (25.5%) and endovascular treatment of AVMs (15.7%). In the case of aneurysm treatment, the spectrum of procedures regularly demonstrated ranged from conventional coiling or balloon-/stent-assisted coiling (18 procedures) to placement of flow diverters (13 procedures).

Technical feasibility of the streaming set-up was assessed by a semiquantitative scale from 1 (poor quality) to 5 (excellent quality). Good or excellent audio perception was reported in 77.5% of all cases (Supplement B).

In 13.7% of cases, fellows experienced a poor audio signal, mainly attributable to slow internet connection speed with delayed voice transmission. Good or excellent video quality was reported in 92.1% of all procedures, and poor quality in only 1%. The handling of the online platform, through which the sessions were streamed and the angiography camera remotely controlled, was found to be good or excellent 94.2% of cases.

**Fig. 1:**
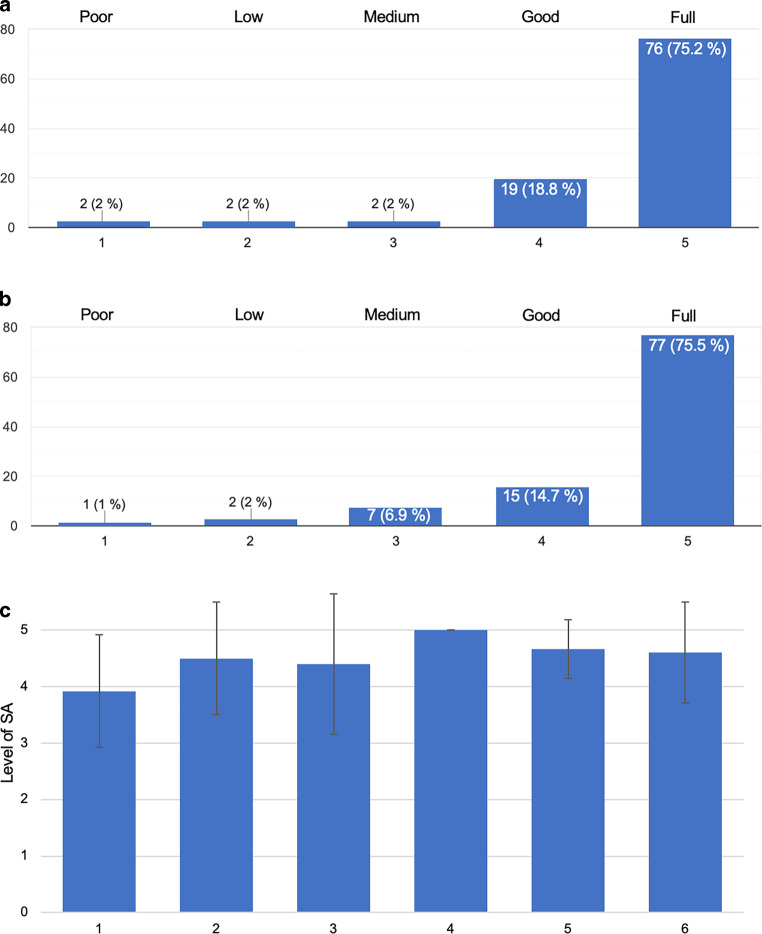
Understandability of the mentor’s descriptions and levels of situational awareness of the fellows. Data were assessed through a semiquantitative 5 level questionnaire with 1 = poor, 2 = low, 3 = medium, 4 = good and 5 = full (y-axis in a, b: absolute number of cases). **a** Reported levels of understanding of the mentors’s descriptions. **b** Reported levels of situational awareness for all cases. **c** The respective mean level (± standard deviation) of situational awareness per fellow

Levels of situational awareness of the fellows were also subjectively assessed through a questionnaire. On the basis of a combined live perception of the interventional procedure and mentor’s moderation using the audiovisual streaming technology, the fellows reported full understanding of the procedural descriptions by the mentors in 75.2%, good understanding in 18.8%, and medium to poor understanding in 6% (Fig. 1a). In line with that, full levels of situational awareness were reported in 75.5% (level 5/5), a good level in 14.7% (level 4/5), and a medium level in 6.9% of cases (level 3/5) (Fig. 1b). Low and poor levels were experienced only in a small proportion of 3% (levels 1–2/5). On an individual basis, the mean level of situational awareness per fellow was 4.5 (± 0.4 SD), with a range from 3.9 to 5 (Fig. 1c).

**Fig. 2:**
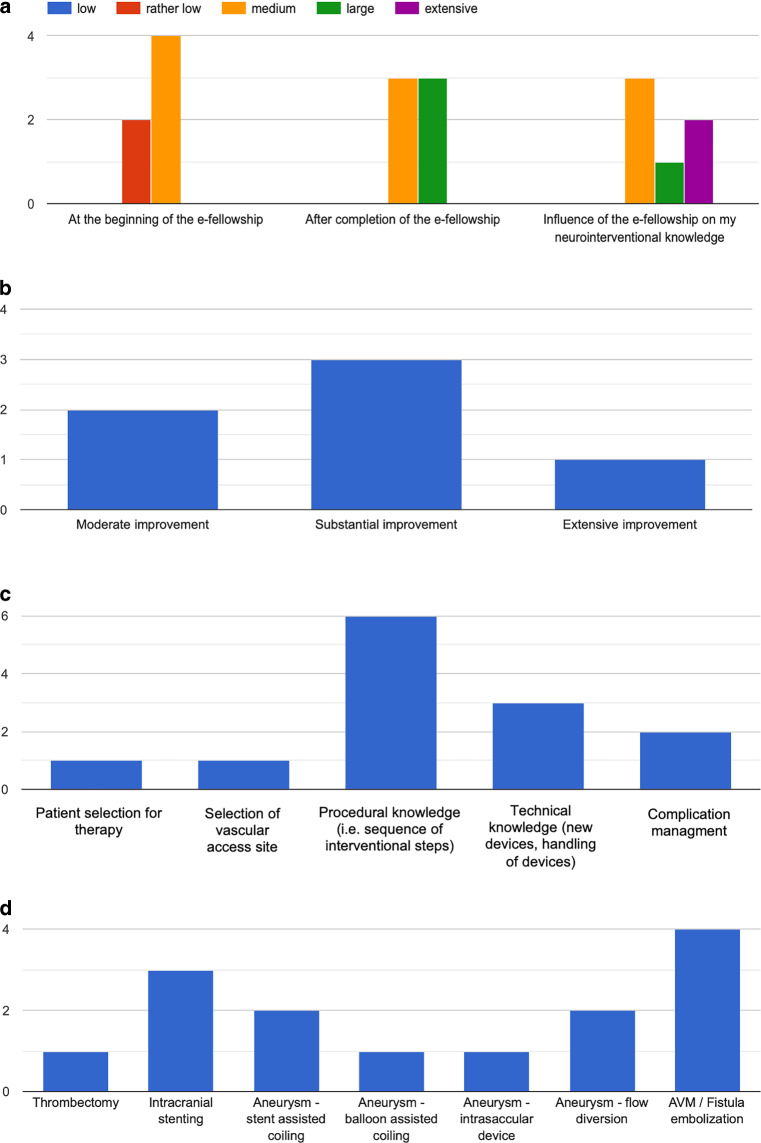
Evaluation of the learning progress through the e‑fellows after completion of the program. Y-axis: number of answers in the questionnaire. **a** Neurointerventional knowledge at different timepoints of the program. **b** Overall learning progress: Reported extent of improvement of neurointerventional knowledge through the fellowship (single answer only). **c** Reported area(s) of knowledge with particular improvement (multiple answers possible). **d** Specific cases with particular learning benefit (multiple answers possible)

While all fellows described their neurointerventional knowledge as rather low (*n* = 2) or medium (*n* = 4) at the beginning, the knowledge after completion of the fellowship was classified as medium or large in three cases each. The influence of the fellowship on their neurointerventional knowledge was reported as medium by half of the participants, as large by one, and as extensive by two participants (Fig. 2a). When asked about the specific fields of knowledge, which improved in particular, all of the fellows stated procedural knowledge (meaning sequence of interventional steps, navigation of vessels, selection of appropriate material, i.e balloon or stent) (Fig. 2c). Half of the fellows also stated technical knowledge (familiarization with new devices, handling of devices). Complication management was also among the repeatedly selected items. On a case level, tele-observership of AVM/fistula treatment was regarded as most educational, followed by intracranial stenting and treatment of aneurysms either by stent-assisted coiling or flow diversion (Fig. 2d). In terms of overall learning progress, a moderate improvement of neurointerventional knowledge was claimed by two fellows, a substantial improvement by three fellows, and extensive improvement by one fellow (Fig. 2b).

Occasional joint sessions for all fellows were organized by initiation of the mentors. Although these were mostly emergency cases with short notice and therefore a regularly full attendance of all fellows not possible, the learning effect was stated to be equal to single sessions by 67% of participants. A total of two participants (33%) stated to profit more from single sessions.

**Fig. 3:**
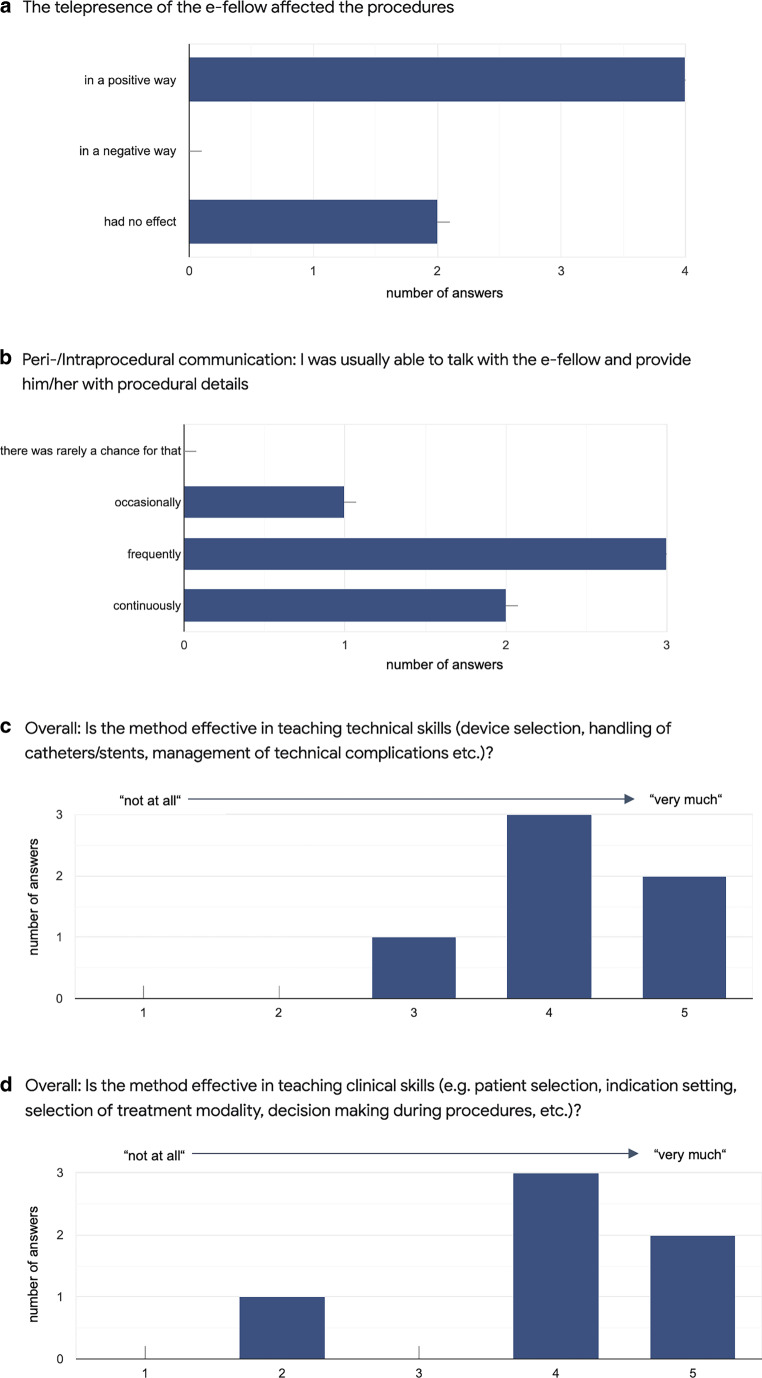
Final course evaluation through the mentors. Use of a five level semiquantitative scale with 1 = “not at all” and 5 = “very much” in (**c**,**d**). **a** Questionnaire: “*The telepresence of the e‑fellow affected the procedures…*” **b** Periprocedural/intraprocedural communication. Questionnaire: “*I was usually able to talk with the e‑fellow and provide procedural details*…”. **c** Questionnaire: “*Overall: is the method effective in teaching technical skills (device selection, handling of catheters, management of technical complications etc.)?*” **d** Questionnaire: “*Is the method effective in teaching clinical skills (e.g. patient selection, indication setting, selection of treatment modality, decision making during procedures, etc.)?*”

After completion of the fellowship and mentorship, the final course evaluation also included an online questionnaire for the mentors (Fig. 3).

Although being exposed to the telestream set-up with the camera rig next to the angiography table, mentors did not report of any relevant technical hindrance of the intervention. Furthermore, the telepresence of the e‑fellow was considered to affect the procedure in a generally positive way in the perception of most mentors (Fig. 3a).

Continuous or at least frequent communication with the fellow including provision of procedural details during the intervention was achievable by 83.3% of the mentors (Fig. 3b). On a five level semiquantitative Likert scale with 1 = “not at all” and 5 = “very much”, the majority of mentors judged the telestream method to be effective in teaching technical and clinical skills (4 or 5 points on the scale in 83.3% of responses) (Fig. 3c, d).

## Discussion

This study systematically assessed the feasibility of a telestream set-up in a remote fellowship environment. In this specific set-up, the tele-observer (e-fellow) followed a neurointerventional case of interest performed by a specialist (mentor) live with real-time video transmission and voice interaction. The mentor provided a one-to-one discussion with the e‑fellow, with the possibility of the fellow to ask questions during the procedure. While previous studies have demonstrated the usefulness of such a system in a scenario in which a less experienced expert performs a neurointerventional case while being continuously proctored by a remotely connected highly experienced specialist, i.e. during treatment of complex aneurysms, the scenario used in this study aims at fostering neurointerventional education by enabling remote in-depth case observation by an e‑fellow. The e‑fellow is assigned to the specialist performing the interventions for a fixed period of time (6 months) with the intention to receive personal mentoring and repetitive remote exposure to specialized cases in a familiar environment.

The spectrum of procedures demonstrated was representative for high volume neurovascular centers, ranging from regular cases such as thrombectomy for acute ischemic stroke to specialized interventions such as flow diversion treatment of an aneurysm or embolization of a dural arteriovenous fistula (Table 2).

Feasibility of this program was evaluated through standardized online questionnaires offered after each telestreamed case. Through this, the majority of cases were evaluated with a response rate of 63%. Missed case evaluations were due to limited time resources of the trainees, who participated in the fellowship parallel to their daily tasks at the respective home hospitals. While full evaluation of all cases would certainly allow an even more representative quantification of case-based learning progress, we decided against asking for a retrospective completion of the forms to avoid recall bias. The final questionnaire after completion of the course aiming at a more comprehensive evaluation achieved a 100% response rate from both the fellows and mentors.

Technical feasibility of a telestreaming set-up largely relies on the quality of the video and audio signal. In > 90% of evaluated cases, video quality was judged to be good or excellent, while satisfactory audio signal was reported in > 85% of cases. The slightly less reliable audio signal might be attributable to the use of different headsets by the fellows with varying quality.

Situational awareness is considered a critical non-technical component of decision-making and can also be applied to complex medical procedures as prerequisite for prevention of complications [12, 13]. In our study, high levels of situational awareness were reported in 75.5% (full level), and 14.7% (good level) of cases. This translates to a satisfactory perception of interventional steps and the projected status of the procedure through the tested audiovisual streaming set-up. Future studies will certainly have to validate these levels of situational awareness in a broader number of users, e.g. through neutral third party observers, rather than through potentially biased user questionnaires.

Although in-person fellowships including hands-on training on patients remains the undisputed standard of basic and advanced neurointerventional training, this set-up might serve as a useful addition to further facilitate training, in particular during times of limited mobility, e.g. during travel restrictions as experienced under the recent COVID-19 pandemic.

Future telestream observerships will ideally include opportunities for e‑fellows to participate regularly in the program with planned or flexible off-time at their place of work. This would allow more convenient access to telestream slots.

### Limitation

Although evaluations were performed anonymously through standardized online questionnaires, a significant desirability bias cannot be ruled out. Future tele-observership programs will therefore preferably include written examinations before and after participation. This would allow to retrieve more objective data on individual improvements of neurointerventional knowledge.

## Conclusion

Based on the evaluations of the remote observers, audiovisual streaming technology is sufficient to gain meaningful situational awareness of neurovascular procedures and is a useful additional tool for neurointerventional training. It facilitates geographically independent training of complex neurointerventional procedures.

## Supplementary Information

**Supplement A:** EYMINT e‑fellowship: Case questionnaire for e‑fellow

**Supplement B:** Technical feasibility of the streaming set-up
