# Supplementary figures and images for: Remote Training of Neurointerventions by Audiovisual Streaming: Experiences from the European ESMINT-EYMINT E-Fellowship Program
Source: Clin Neuroradiol. 2022 Jul 13;33(1):137–45. doi: 10.1007/s00062-022-01192-9 (PMC9277595; doi:10.1007/s00062-022-01192-9)

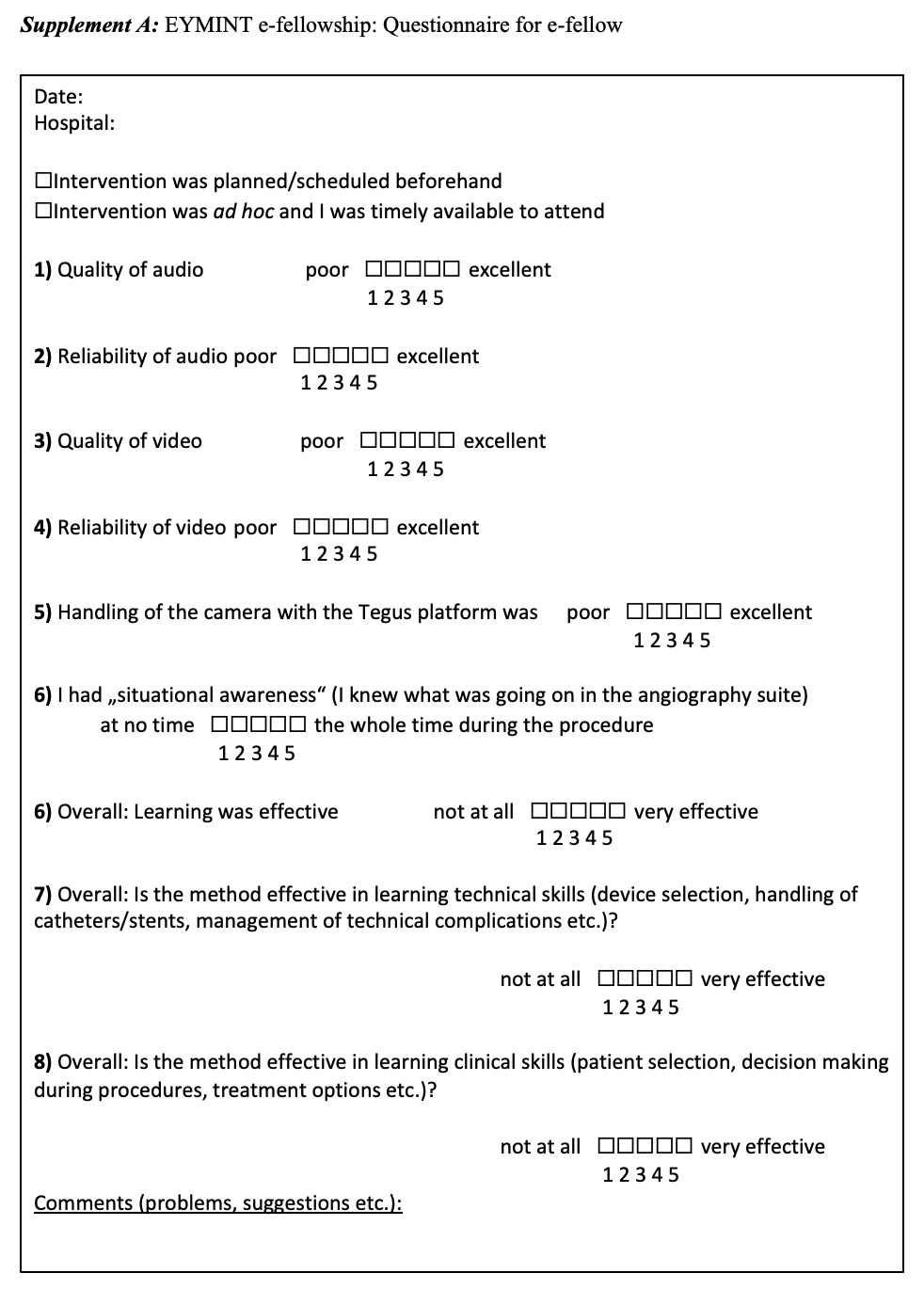

Supplement: Supplementary file 1 — Supplement A: EYMINT e‑fellowship: Case questionnaire for e‑fellow [file 62_2022_1192_MOESM1_ESM.tiff]

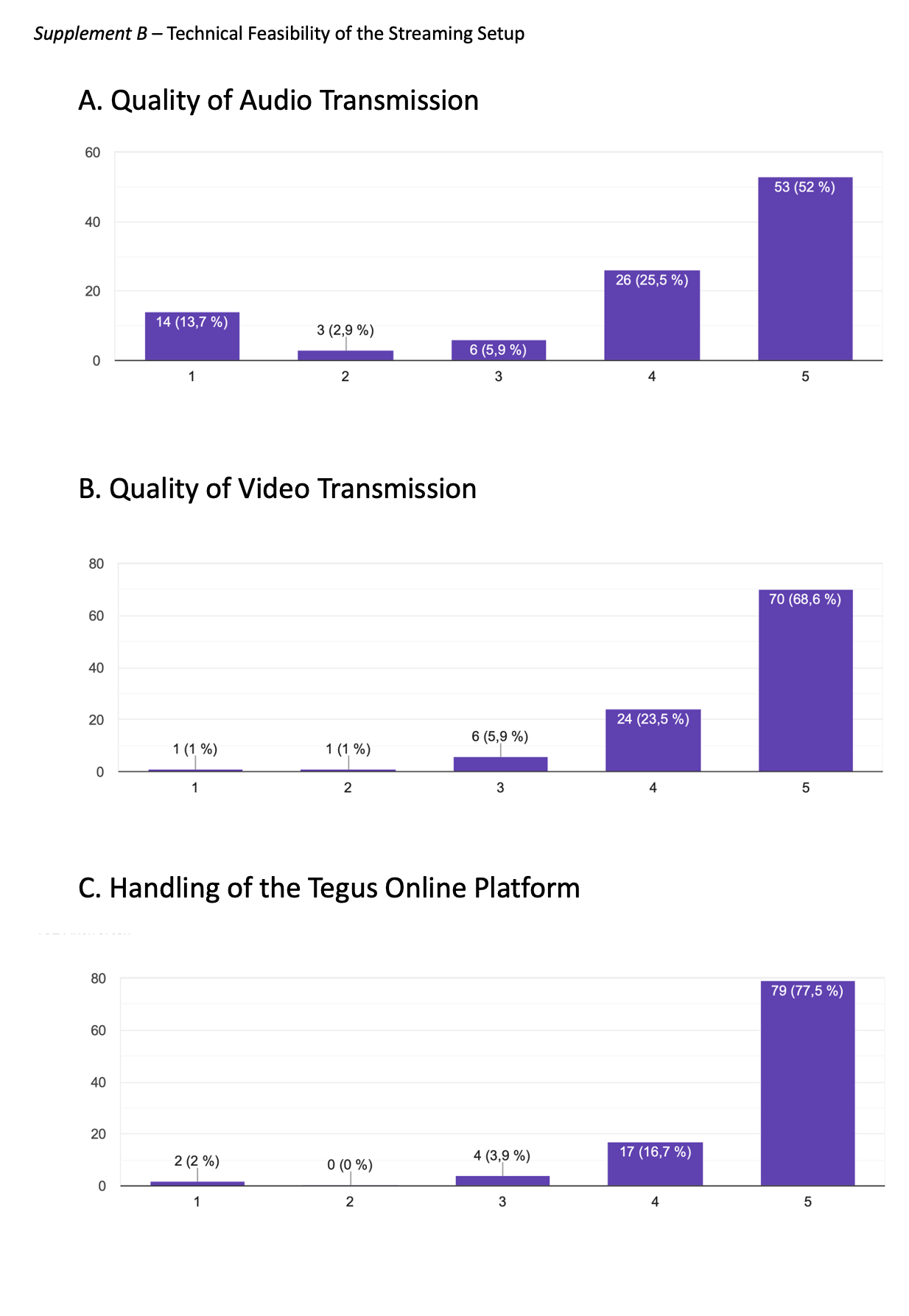

Supplement: Supplementary file 2 — Supplement B: Technical feasibility of the streaming set-up [file 62_2022_1192_MOESM2_ESM.tiff]
